# Health Tract, No. 231

**Published:** 1865

**Authors:** 


HEALTH TRACT, No. 231.

### DRUGS AND DRUGGERY.

The longer an experienced physician lives, the more value he places on medicine as an agent for the removal of disease; at the same time the less and less is he inclined to give medicine except in urgent cases, prefering to let nature put forth her energies to throw off the disease while he stands by and watches; and if she is about to fail, then he helps at the nick of time by the faithful drug, knowing that there are cases where neither nature nor medicine could cure separately, but when their united power at the critical juncture is all-efficient to save a loved one from a premature and unnecessary grave. Blessings be upon every experienced, observant, and faithful physician! for he deserves, everywhere, the confidence, the respect, and the warm attachment of every well-informed mind.

But the question is: How does a physician know what and when to give? Observation and judgment are his guides, amounting in some cases to a degree of infallibility. In one respect an educated physician of long practice is as different from the multitude as day is from night. Most persons give their confidence to a remedy from a single observation of its efficiency, while, if it succeeds in several additional cases, he is carried away in his admiration of its powers, and before he is aware of it, he is recommending it to every body, and for every thing, and often goes so far as to advise those who are perfectly well to take it as a means of preventing all sickness; thus it is that "popular remedies," as they are called, "have their day,"

"And straight are seen no more"!

The greatest vulgar enthusiast find his faith to waver in his favorite remedy on the very first failure; and when that fails, he has no other resource and is powerless as an infant, because he has no idea of the principles of action; this is real, pure, unsophisticated quackery. The true physician is not elated by one success, or a dozen, or a hundred, nor is he depressed by as many failures, because he has formed his opinions from the mass of evidence, and not from single cases; in addition he knows the principles of action of all his remedies, and when one fails for want of power, or imperfection, or deterioration of materials, he takes some other remedy of similar modes and powers of action, and is constantly applying general principles, just as a good lawyer can apply a principle to a case whose like never appeared before; like a good classical scholar who can tell the meaning of a word the first time he ever saw or heard it, whether of Greek or Latin origin. The physician may not be able to tell why a medicine acts so and so, but he knows the fact of its thus acting, as certain as his own existence, simply from repeated observations. Every thing taken internally as a remedy for a symptom, except natural food and drink, is a drug or medicine in the true sense of the word, however familiar the article may be, whether it be sea-water or tartar emetic, ashes, or strychnine; and he who decries medicine is an unmitigated ass, a consummate fool.

